# BGD: A Database of Bat Genomes

**DOI:** 10.1371/journal.pone.0131296

**Published:** 2015-06-25

**Authors:** Jianfei Fang, Xuan Wang, Shuo Mu, Shuyi Zhang, Dong Dong

**Affiliations:** Institute of Molecular Ecology and Evolution, SKLEC & IECR, East China Normal University, Shanghai, China; Université Paris-Sud, FRANCE

## Abstract

Bats account for ~20% of mammalian species, and are the only mammals with true powered flight. For the sake of their specialized phenotypic traits, many researches have been devoted to examine the evolution of bats. Until now, some whole genome sequences of bats have been assembled and annotated, however, a uniform resource for the annotated bat genomes is still unavailable. To make the extensive data associated with the bat genomes accessible to the general biological communities, we established a Bat Genome Database (BGD). BGD is an open-access, web-available portal that integrates available data of bat genomes and genes. It hosts data from six bat species, including two megabats and four microbats. Users can query the gene annotations using efficient searching engine, and it offers browsable tracks of bat genomes. Furthermore, an easy-to-use phylogenetic analysis tool was also provided to facilitate online phylogeny study of genes. To the best of our knowledge, BGD is the first database of bat genomes. It will extend our understanding of the bat evolution and be advantageous to the bat sequences analysis. BGD is freely available at: http://donglab.ecnu.edu.cn/databases/BatGenome/.

## Introduction

Bats are mammals of the order Chiroptera, representing about 20% of all classified mammalian species worldwide [1]. Bats have long been regarded as one of the most unusual and specialized animals. They have long been regarded as special animals for the sake of being mysterious flyers of the night, and they are actually the only mammalian group with true flight capability. Furthermore, most of the bats are masters of echolocation, which allows bats to detect, localize, and even classify their prey in the complete darkness.

**Fig 1:**
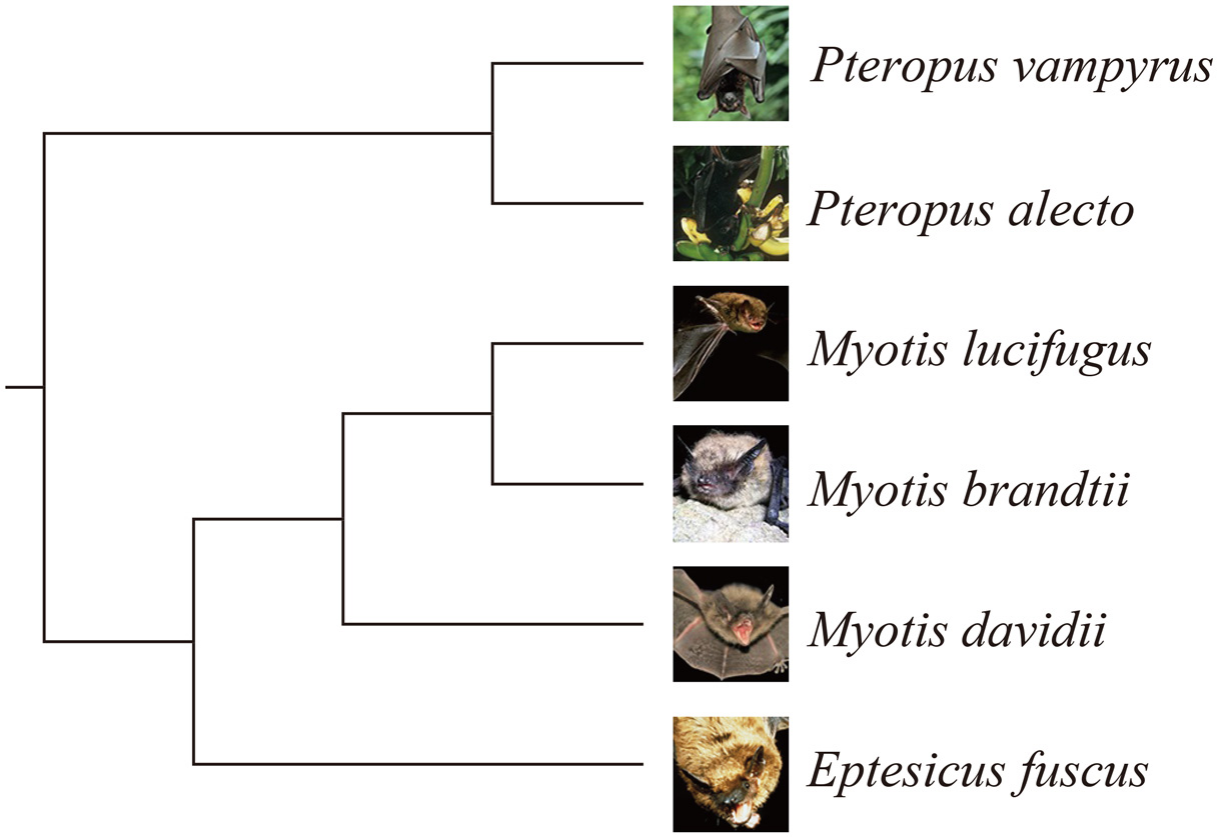
Phylogenetic tree of bat species involved in BGD.

For the sake of these specialized phenotypic traits, many researches have been devoted to explore the underlying molecular mechanisms of bats at the sequence level [2]. For example, the ‘hearing gene’ *Prestin* was recently reported to have undergone sequence convergence between echolocating bats and dolphins [2]. Energy metabolism genes were reported to be targets of natural selection and allowed adaptation to the energy demand during the origin of flight [3]. Recently, several bat genomes have been sequenced and assembled, and these data provided us valuable resources for further scientific researches on the biology and conservation of bats [4–6]. The prevailing theory is that flying vertebrates (bats and birds) tends to have smaller genomes than other vertebrates due to metabolic constraints on cell sizes and genome sizes [7]. Consistent with this finding, the bat genomes (~2 Gb) are relatively smaller than other mammals. Up to date, there’s no specialized and comprehensive database that focuses on storage of bat genomes. To conveniently access the bat genomes, a uniform database for the bat genomes is necessary. In this work, we collected the genome sequences of six bats (including two megabats, *Pteropus alecto*, *Pteropus vampyrus* and four microbats, *Myotis davidii*, *Myotis brandtii*, *Myotis lucifugous*, *Eptesicus fuscus*, Fig 1) from various databases, and uniformly *in silico* annotated these genome sequences. BGD was developed as a public database for readily accessing the bat genomes and genes, and a platform for extensive biological interpretations.

## Materials and Methods

The genome and gene sequences of *Pteropus vampyrus* (Ptevap1.0) and *Myotis lucifugous* (Myoluc2.0) were downloaded from Ensembl database (http://www.ensembl.org/index.html) [8], and other four bat genomes (*Pteropus alecto*, ASM32557v1; *Myotis brandtii*, ASM41265v1; *Myotis davidii*, ASM32734v1; *Eptesicus fuscus*, EptFus1.0) were downloaded from NCBI genome database (http://www.ncbi.nlm.nih.gov/genome/). All the genomes have not been assembled into chromosomes, and sequence scaffolds were obtained. Accurately prediction of protein-coding genes is the most important task of genome annotation. The bat genomes were sequenced separately, and bat genes were annotated using different pipelines. For example, the genes of *Pteropus vampyrus* and *Myotis lucifugous* were predicted based on homology searching method in Ensembl database. Moreover, the gnome of *Eptesicus fuscus* is still not well annotated. So, a uniform pipeline is necessary for gene annotation. In this work, we uniformly annotated the bat genomes using a combination of homology-based and *de novo* method according to previously published pipeline [4]. Because human, mouse and dog proteins are well annotated in mammals. For the homology-based method, human, mouse and dog proteins were collected and mapped on the genomes using tblastn. Then, homologous genome sequences were aligned against the matching proteins using Genewise (version 2.2.0) [9]. For the *de novo* prediction method, Augustus [10] and Genescan v1.0 [11] were employed. The RNA-seq data of *Myotis davidii*, *Myotis brandtii and Pteropus alecto* were also downloaded to help annotate the genomes. Finally, all lines of evidences were combined together using EVM (r2012-06-25) software (evidence_modeler.pl --genome bat_genome.fa --gene_predictions --weights./weight.txt \ --protein_alignments./bat_genblastg.gff --transcript_alignments \--exec_dir 50 \). At last, 21237, 16956, 21593, 22125, 19496, 18366 protein coding genes were obtained from the genomes of *Pteropus alecto*, *Pteropus vampyrus*, *Myotis davidii*, *Myotis brandtii*, *Myotis lucifugous*, *Eptesicus fuscus*, respectively. We compared our predicted genes with previous annotated genes, and the result showed that these results are very similar (S1 Fig). Then, a serious of annotation works were performed in order to obtain comprehensive genomic functional information. First, the prediction of gene function domains was performed using InterproScan v5 [12] software against InterPro database [13], which integrates together predictive information about protein function from a number of resources and provides an overview of protein functions. Second, full-length cDNA sequences of bats were mapped to genomes using BLAT [14]. Then, we performed BLASTP (E-value 1e-5) against NCBI RefSeq and UniRef databases to find the best hit for each gene. The statistics of six bat genomes and annotated information are shown in Table 1.

**Table 1:** Statistics of six bat genomes.

Species	Number of scaffolds	Scaffold N50 (bp)	Number of contigs (bp)	Contig N50 (bp)	Number of genes
*Pteropus vampyrus*	96,944	124,060	388,808	8,527	16,956
*Pteropus alecto*	65,598	15,954,802	170,164	31,841	21,237
*Myotis brandtii*	169,750	3,225,832	325,414	23,289	22,125
*Myotis lucifugus*	11,654	4,293,315	72,785	64,330	19,496
*Myotis davidii*	101,769	3,454,484	325,280	15,182	21,593
*Eptesicus fuscus*	6,789	13,454,942	167,058	21,392	18,366

## Results and Discussion

**Fig 2:**
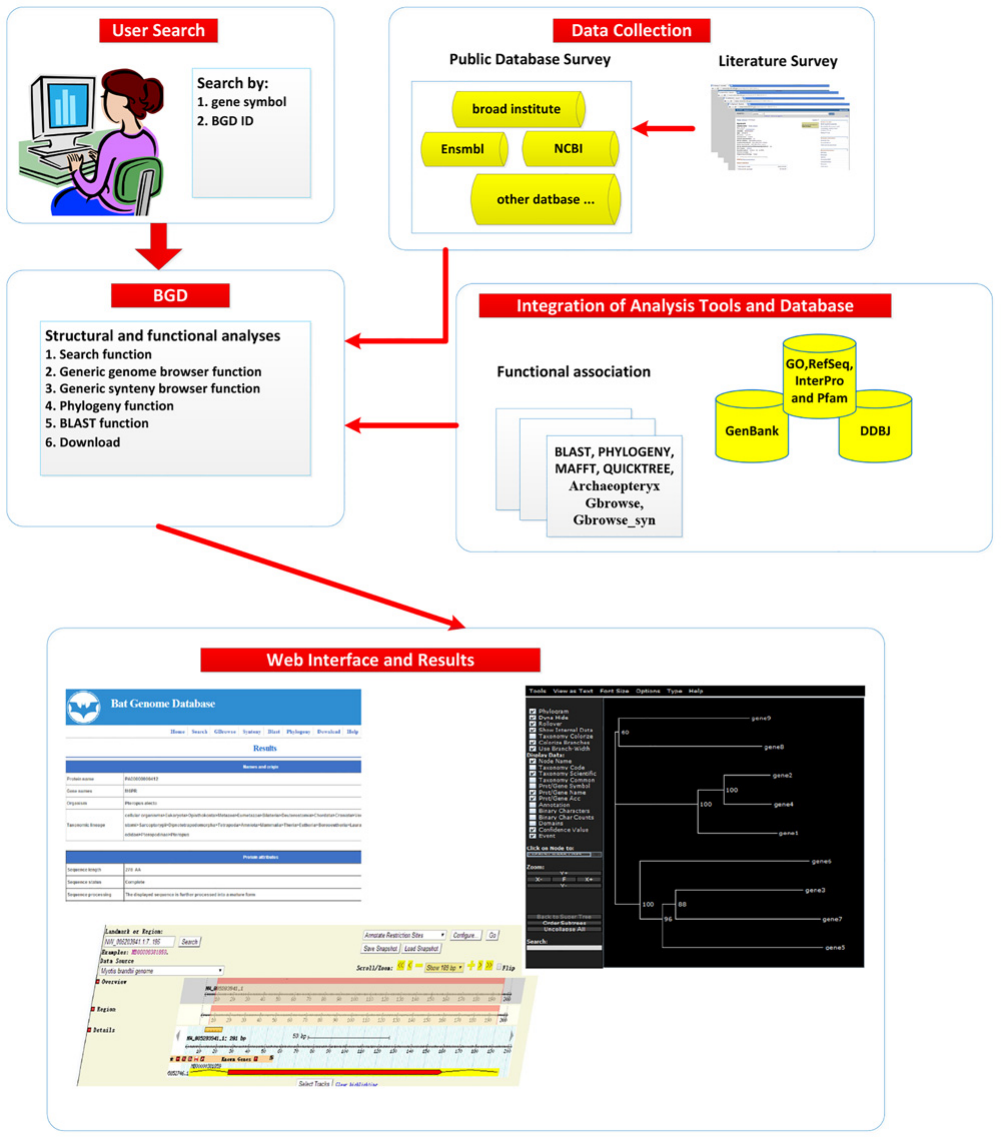
System flow of BGD.

We stored and managed data for BGD using MySQL on a Linux system. BGD uses several common gateway interface scripts to process user’s input to search the database. A schematic diagram of BGD organization is shown in Fig 2.

### Retrieve data

The searching engine can be used to acquire the annotated gene information. In the current version, BGD has been designed with simple search and batch search engines and can be accessed with gene symbols or BGD ID. BGD can return a list of bat genes, coupled with biological implications, Gene ontology information and nucleotide or amino acid sequences.

### Genome browser

BGD utilizes a genome browser, implemented with GBrowse v2.0 [15], to navigate gene annotation along the bat genome assemblies. GBrowse is a well-known browser that combines database and interactive web pages to display the annotation of the genome. The gnome browser connecting to a MySQL backend is used, and researchers can view the genomic features aligned to the genome.

### Synteny browser

A six-way genomes comparison among the bat species was performed, and we used OrthoCluster v1 [16] for the detection of synteny blocks among bat genomes. The result can be visualized using GBrowse (version 1.69). It can be used to compare co-linear regions of multiple genomes using the familiar GBrowse-style web page. The ‘hearing gene’ *Prestin* was recently widely reported in bats [2, 17], which play important role in bat echolocation. Here, we showed an example of comparative synteny of *Prestin* gene (S2 Fig) in BGD synteny browser.

### Phylogeny server

To better understand the evolution of bat genes, BGD provides an online phylogeny tool. Considering the accuracy and efficacy, neighbor-Joining method was implemented, and only 50 or 100 bootstrap replicates can be selected. In the current version, BGD have employed phyloXML (version 1.10) software [18] for the online phylogenetic tree visualization. Mozilla Firefox or Safari web browser are highly recommended, and Sun Java 1.5 or higher version is needed. An example of *Prestin* genes were provided in BGD, and the online result was shown in S3 Fig.

### BLAST server

BLAST is one of the most useful entrance site for genome database. At BGD, researchers can search against a variety of genomic sequences. We packed all bat gene sequences to facilitate search for homologs of other mammalian species.

### Future directions

Other bat genome sequences and population genomic studies for bats should be forthcoming. It will be very useful for analyzing bat genome and gene sequences to explore the bat evolution. Future directions include an incorporation of more bat genome data to provide a richer source of comparative implementation of bat sequence analysis.

## Conclusion

We presented an easily accessible database, offering access to the genome of bat species. The integration of annotated genome can enhance the role of BGD as an essential resource for bat evolution analysis. The BGD enables use of genomic data toward facilitating further understanding of the fundamental biology of bat species, and the adaptation of specialized traits. To the best of our knowledge, BGD is the first repository centralizing the genomes and genes of bat species. The database not only provides a large resource for the bat researches, but also supplies a platform for comparative genomic analysis.

## Supporting Information

S1 Fig Comparison of identified genes between our findings and original results. (JPG)

S2 Fig Comparative synteny of *Prestin* gene in bat genomes. (JPG)

S3 Fig Phylogenetic tree of *Prestin* gene generated in BGD database. (JPG)
