# Supplementary figures and images for: BGD: A Database of Bat Genomes
Source: PLoS One. 2015 Jun 25;10(6):e0131296. doi: 10.1371/journal.pone.0131296 (PMC4482021; doi:10.1371/journal.pone.0131296)

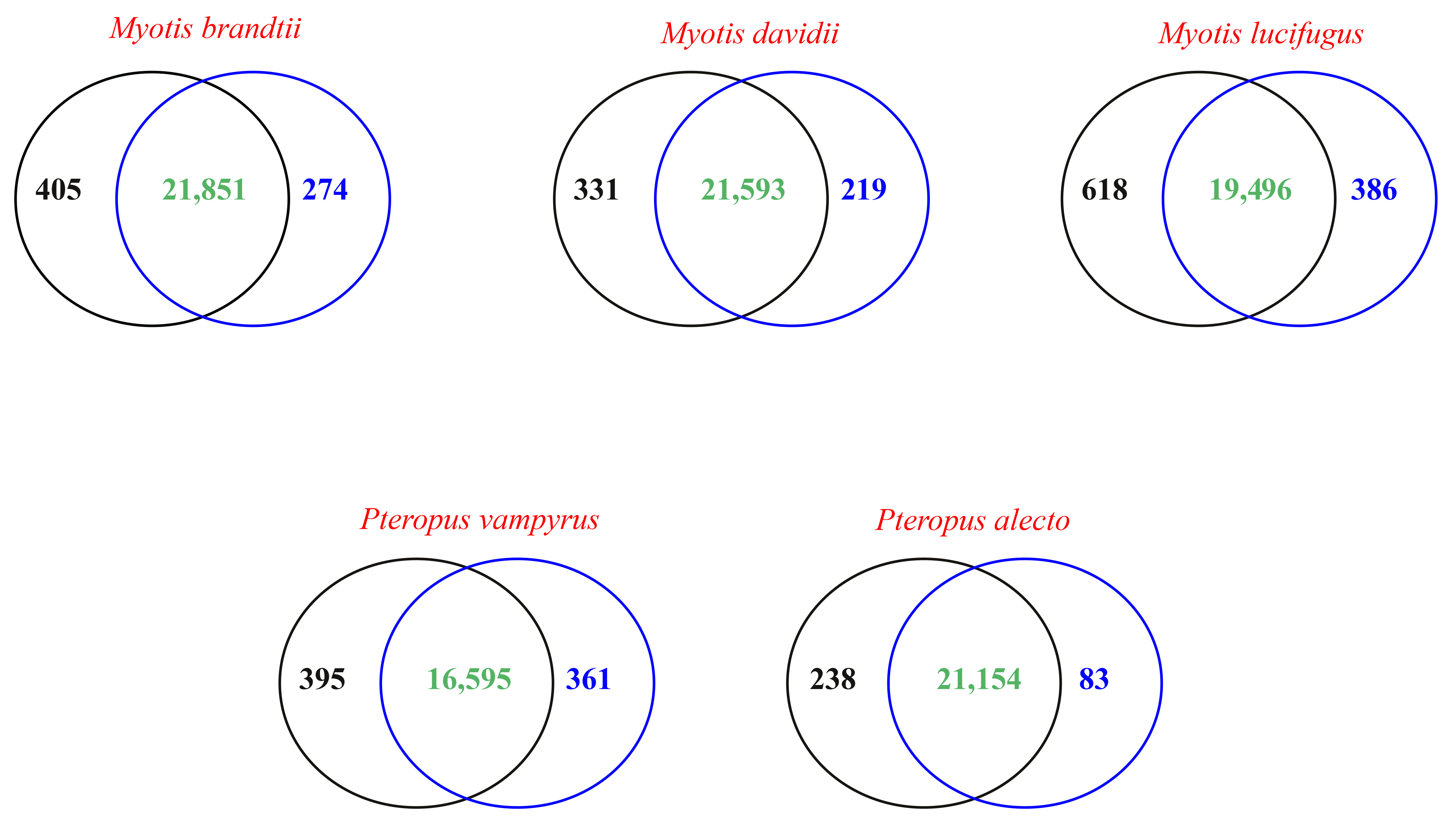

Supplement: S1 Fig — (JPG) [file pone.0131296.s001.jpg]

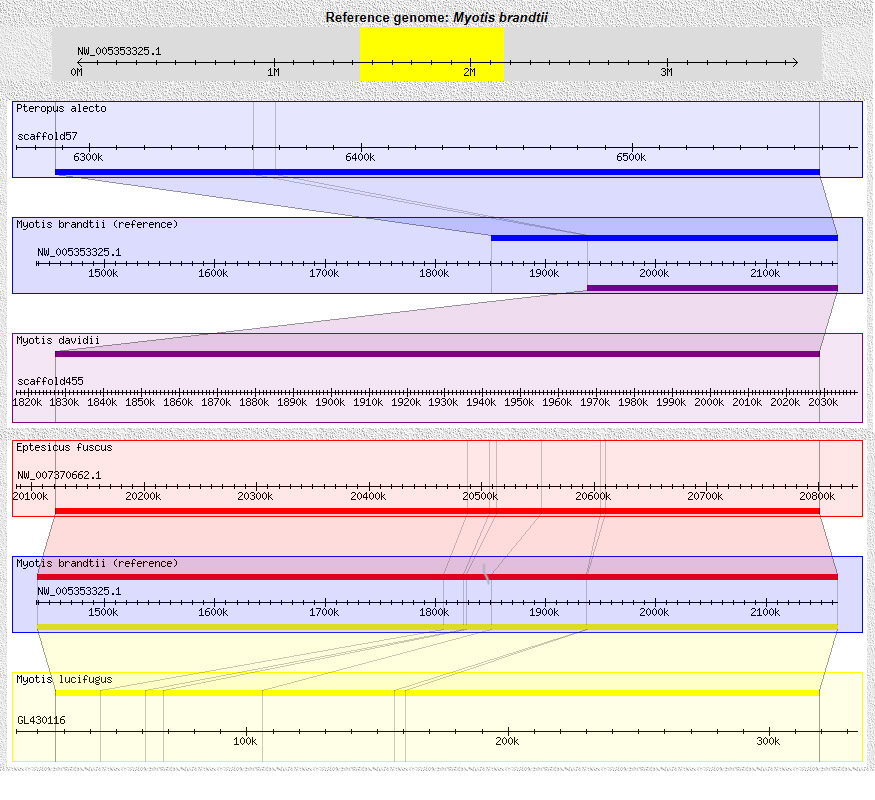

Supplement: S2 Fig — (JPG) [file pone.0131296.s002.jpg]

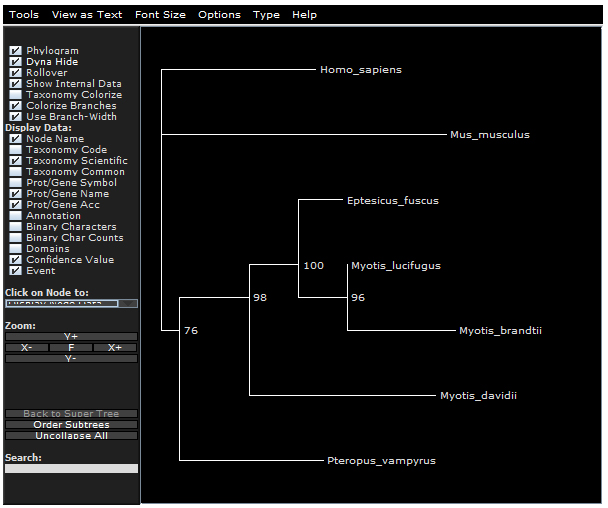

Supplement: S3 Fig — (JPG) [file pone.0131296.s003.jpg]
